# Angiopoietin-like 8 (ANGPTL8) as a potential predictor of NAFLD in paediatric patients with Prader-Willi Syndrome

**DOI:** 10.1007/s40618-020-01444-w

**Published:** 2020-10-16

**Authors:** C. Mele, A. Crinò, D. Fintini, S. Mai, A. Convertino, S. Bocchini, P. Di Paolo, G. Grugni, G. Aimaretti, M. Scacchi, P. Marzullo

**Affiliations:** 1 Division of Endocrinology, Department of Translational Medicine, University of Piemonte Orientale, Novara, Italy; 2 Division of General Medicine, Istituto Auxologico Italiano, IRCCS, San Giuseppe Hospital, Piancavallo, Verbania, Italy; 3 Reference Center for Prader-Willi Syndrome, Bambino Gesù Children’s Hospital, Research Institute, Palidoro (Rome), Italy; 4 Laboratory of Metabolic Research, Istituto Auxologico Italiano, IRCCS, San Giuseppe Hospital, Piancavallo, Verbania, Italy; 5 Radiology Unit, Bambino Gesù Children’s Hospital, Research Institute, Palidoro (Rome), Italy; 6 Division of Auxology and Metabolic Diseases, Istituto Auxologico Italiano, IRCCS, San Giuseppe Hospital, Piancavallo, Verbania, Italy

**Keywords:** ANGPTL8, Prader–Willi syndrome, NAFLD

## Abstract

**Purpose:**

Angiopoietin-like 8 (ANGPTL8) is a liver- and adipose tissue-produced protein that predicts non-alcoholic fatty liver disease (NAFLD) and altered metabolic homeostasis in the general population as well as in persons with common and genetic obesity, including the Prader–Willi syndrome (PWS). However, its metabolic correlate in paediatric patients with respect to PWS is unknown.

**Methods:**

This cross-sectional study investigated circulating ANGPTL8 and adipocytokines levels in 28 PWS and 28 age-, sex- and BMI-matched children and adolescents (age, 7.0–17.8y) in relation to NAFLD and metabolic homeostasis assessed by OGTT, paediatric metabolic index (PMI) and fatty liver index (FLI), liver ultrasonography (US), as well as dual-energy X-ray absorptiometry (DEXA) for analysis of fat (FM) and fat-free mass (FFM).

**Results:**

At the set level of significance, PWS children showed lower values of FFM (*p* < 0.01) but healthier insulin profiles (*p* < 0.01) and PMI values (*p* < 0.05) than matched controls. By US, the prevalence of NAFLD was similar between groups but less severe in PWS than controls. Analysis of ANGPTL8 levels showed no difference between groups, yet only in PWS ANGPTL8 levels were associated with ALT levels, FLI values and NAFLD. In stepwise multivariable regression analysis on merged data, ANGPTL8 levels were independently predicted by BMI SDS, leptin levels and NAFLD.

**Conclusion:**

ANGPTL8 levels are similar in PWS and controls and, overall, they are directly associated with the presence and severity of NAFLD in patients with PWS.

## Introduction

Prader-Willi syndrome (PWS) represents one of the most common form of genetic obesity with an estimated incidence rate of 1 in 25,000 live births [1, 2]. It is an imprinted neurobehavioral condition caused by the lack of expression of genes located on the paternal chromosome 15q11.2-q13. Three main genetic subtypes have been detected in PWS: 15q11-q13 deletion (65–75% of cases), maternal uniparental disomy of chromosome 15 (UPD15) (20–30% of cases), and imprinting defects, chromosomal translocation or rearrangements of the 15q11-q13 region (1–3%) [3]. Clinically, the dominant features of PWS include neonatal hypotonia with poor sucking and failure to thrive, mildly dysmorphic acro-facial features, kyphoscoliosis, autonomic dysregulation and a variable number of endocrine disorders comprising short stature and hypogonadism [4]. Cognitive impairment usually presenting as mild intellectual disability, may coexist with developmental neuropsychomotor delay and behavioral disturbances, with a few patients developing psychiatric disorders [5].

PWS is typically associated with a lack of satiety due to hypothalamic dysfunction, which generates obsessive craving for food approximately by the age of 2 years and progresses to morbid obesity unless strict parental surveillance on access to food is adopted [6]. Changes in body composition in patients with PWS develop even before hyperphagia occurs [7] and result in higher fat mass (FM) and predominant accumulation of subcutaneous fat, lower fat-free mass (FFM) and greater ratio of intramuscular adipose tissue/skeletal muscle [8], impaired muscle function [9] and decreased excitability of cortical motor areas [10] when compared to controls. In terms of energy homeostasis, the voluntary activity is decreased in PWS and is associated with low values of resting energy expenditure (REE), although this defect seems proportional to lower values of lean body mass [11].

Despite this adverse phenotype, metabolic impairment in young patients with PWS is generally milder than in controls matched for BMI due to preferentially peripheral fat accumulation and heightened insulin sensitivity [12, 13]. In the past several years, increasing interest has focused on non-alcoholic fatty liver disease (NAFLD) as a marker of metabolic dysfunction in children and adolescent with obesity [14, 15]. In the youth, the metabolic and hypoxic background associated with NAFLD, i.e. hyperglycemia, insulin resistance, dyslipidemia and obstructive sleep apnea (OSA), predisposes to the typical spectrum of liver disorders extending from liver steatosis to non-alcoholic steatohepatitis (NASH), with further potential advancement to fibrosis and/or cirrhosis [16]. In children or adolescents [17] as well as in adults with PWS [18], however, the risk and severity of NAFLD is lower than controls, although the determinant/s for this discrepancy remain unclear. Liver produces several hepatokines and acts in humans as the major production site of angiopoietin-like 8 (ANGPTL8) [19], which is involved in different metabolic pathways relating to glucose and lipid metabolism as well as liver function [20, 21]. Like other members of the angiopoietin family, ANGPTL8 is able to reduce serum triglyceride clearance through the inhibition of lipoprotein lipase (LPL), which hydrolyzes triglycerides from VLDL lipoproteins and chylomicrons into fatty acids and glycerol, thus allowing for their subsequent intracellular beta-oxidation (skeletal and cardiac muscle) or re-synthesis into triglycerides (adipose tissue) [22]. Evidence has recently accumulated on ANGPTL8 as a potential marker of NAFLD, metabolic dysfunction and the hypoxic damage related to obstructive sleep apnea syndrome (OSAS) [23–25].

In a previous study on adults with PWS, ANGPTL8 levels were found to be lower in patients with PWS than in controls matched for BMI and to parallel the severity of NAFLD [18].

To date, the metabolic significance of ANGPTL8 in childhood and adolescence and, particularly, in children with PWS is unknown. As such, this study was designed to investigate ANGPTL8 levels in children with PWS and controls matched for BMI SDS in association with metabolic homeostasis, markers of adiposity and NAFLD.

## Methods

### Patients

This study enrolled 56 patients, consisting of 28 children and adolescents with PWS and 28 controls matched for age, sex and BMI SDS. Patients with PWS were referred to the Center for Prader–Willi Syndrome of the Bambino Gesù Children’s Hospital in Rome, Italy, where diagnosis was based on typical syndromic features and molecular genetic studies of chromosome 15. The genetic analysis revealed 15q11-q13 deletion in 17 patients (9 males and 8 females) and UPD15 in 11 patients (8 males and 3 females). With respect to hormone replacement therapy, 19 patients with PWS were treated and 9 patients had previously undergone growth hormone (GH) treatment (mean dose 0.16 ± 0.04 mg/kg/week), while 2 patients with PWS and one subject of the control group were treated with levothyroxine. None of patients or controls were treated with gonadal steroids. Four PWS patients were treated with Continuous Positive Airway Pressure (cPAP) for OSAS. For all study participants, exclusion criteria included previously known liver disease, kidney failure, autoimmune diseases, uncontrolled hypothyroidism and/or diabetes mellitus, chronic exposure to anti-inflammatory steroids. Moreover, patients on medication that interfere with liver function or with a known propensity to favour the development of microvesicular steatosis were excluded from the present study. Alcohol consumption was investigated, and none was an alcohol drinker.

The study design was conformed to the ethical guidelines of the Declaration of Helsinki and was firstly approved by the Ethical Research Committees of the Bambino Gesù Children’s Hospital. Written informed consent was obtained from all participants by their parents, and from patients, when appropriate. The study protocol was conformed to the guidelines of the European Convention on Human Rights and Biomedicine concerning biomedical research.

### Body measurements

Weight and height were measured to the nearest 0.1 kg and 0.1 cm, respectively, using standard methods. Waist circumference (WC) was measured midway between the lowest rib and the top of the iliac crest after expiration; hip measurements were taken at the greatest circumference around the nates. BMI was expressed as body mass (kg)/height (m)^2^, and BMI SDS was calculated according to BMI reference tables [26]. The BMI cut-off point of ≥ 2 SDS was used to define obesity [26]. Pubertal development was assessed according to Tanner’s criteria [27].

A dual-energy X-ray absorptiometry (DEXA; Hologic Inc., Bedford, MA) was performed by the same operator for the assessment of body mass, FFM, FM and trunk fat mass (TFM). FFM/FM and trunk fat/FM ratios were also calculated.

In order to assess the presence and severity of NAFLD, liver ultrasonography (US) was performed using a high-resolution US system (LOGIQ 7, GE Healthcare, Waukesha, WI, USA) by the same operator who was blinded to clinical data. The degree of NAFLD was assessed semi-quantitatively on the basis of hepatorenal echo contrast, liver brightness, deep attenuation and vascular blurring. NAFLD was established by a validated method of US grading (categorized as: G0 = absent; G1 = mild; G2 = moderate, G3 = severe steatosis) [28].

### Metabolic studies

Glucose homeostasis was evaluated by fasting glucose levels, OGTT-derived glucose and insulin levels at time 0 and 120 min, and glycated haemoglobin (HbA1c) levels in all subjects. Glucose tolerance was assessed according to ADA guidelines for children and adolescents [29]. Insulin resistance was calculated by the homeostatic model of insulin resistance (HOMA-IR) index: insulin (mIU/L) × [glucose (mmol/L)/22.5] [30].

Metabolic risk was assessed through the paediatric metabolic index (PMI), an algorithm that has been shown to correlate with visceral fat adiposity, preperitoneal fat thickness, transaminases, fatty liver and insulin resistance in a large population of children aged between 5 and 17 years [31]. PMI is expressed as: PMI = WC / ( – 0.02 * BMI^2^ + 3.67 * BMI + 3.24) * (TG / 0.88) * (1.32 / HDL) (for female < 10 years), WC / ( – 0.02 * BMI^2^ + 3.67 * BMI + 3.24) * (TG / 1.04) * (1.34 / HDL) (for female ≥ 10 years), WC / ( – 0.02 * BMI^2^ + 3.62 * BMI + 3.72) * (TG / 0.77) * (1.38 / HDL) (for male < 10 years), WC / ( – 0.02 * BMI^2^ + 3.62*BMI + 3.72) * (TG / 1.06) * (1.30 / HDL) (for male ≥ 10 years). A PMI value greater than 1.7 is considered as suggestive of an unfavourable metabolic profile in terms of lipid profile, liver function and insulin resistance.

Biochemical components of routine analysis were also used to derive the fatty liver index (FLI), an algorithm previously developed to assess the degree of fatty liver [32] and expressed as: (e^[0.953 × ln(TG) + 0.139 × BMI + 0.718 × ln(GGT) + 0.053 × WC − 15.745]^ / (1 + ^e[0.953 × ln(TG) + 0.139 × BMI + 0.718 × ln(GGT) + 0.053 × WC − 15.745]^) × 100, with triglycerides measured in mg/dl (1 mg/dl = 0.01129 mmol/l), gamma-glutamyl transpeptidase (GGT) in U/l, and WC in cm. The FLI score range is 0–100.

### Laboratory tests

Blood samples were drawn at 08.00 a.m. under fasting conditions, then vials were centrifuged, and sera were stored at − 80 °C until assay.

Serum ANGPTL8 levels were assessed using a commercially available human EIA kit (Phoenix Pharmaceutics, Inc, Burlingame, CA, USA). The assay procedure was performed in accordance with the manufacturer’s instructions. All samples were analyzed in duplicate. Intra-assay CV and inter-assay CV of ANGPTL8 were less than 10% and 15% respectively. Minimum detectable concentration was 0.12 ng/mL. This EIA is specific for human ANGPTL8, and quality controls were included in all EIA measurements with the results falling within the expected range.

Serum adiponectin levels were determined by an enzyme-linked immunosorbent assay (DRG Instruments GmbH, Marburg, Germany), the detection limit was 1.56–100 ng/ml, sensitivity was 0.2 ng/ml, inter- and intra-assay CV was 2.4–8.4 and 0.9–7.4%, respectively. Serum leptin concentrations were quantified using a commercially available ELISA kit (Mediagnost GmbH, Reutlingen, Germany) with sensitivity of 0.2 ng/ml as well as inter- and intra-assay CVs of 6.8–8.3% and 5.5–6.9%, respectively.

Routine laboratory data included levels of aspartate aminotransferase (AST), alanine aminotransferase (ALT), GGT, glucose, total cholesterol (CHO), high-density (HDL) and low-density lipoprotein (LDL) cholesterol, triglycerides (TG) and HbA1c, measured by enzymatic methods (Roche Diagnostics, Mannheim, Germany). Levels of insulin were measured using a Cobas Integra 800 Autoanalyzer (Roche Diagnostics, Indianapolis, IN, USA). A two-site, solid-phase chemiluminescent immunometric assay or competitive immunoassay (Immulite 2000 Analyzer; DPC, Los Angeles, CA) was used to determine C-peptide levels. Total IGF-I levels were assayed by chemiluminescence IGF-I immunoassay by Liaison (Nichols Advantage, San Juan Capistrano, CA), having a sensitivity of 6 µg/litre, intra-assay and inter-assay CVs of 4.8 and 6.7%, respectively.

### Data analysis

Statistical analysis was performed using SPSS version 21 (Somers, NY, USA). Values are expressed as means ± standard deviation (SD) or percentage. Data points not normally distributed obtained by the Shapiro–Wilk test were log-transformed to improve the symmetry and homoscedasticity of the distribution. For comparative analysis, ANOVA between the 2 groups was used. Pearson’s correlation analysis and the Chi square were used to identify significant associations between variables of interest. Stepwise multivariable regression analysis was used to evaluate the independent association of ANGPTL8 with metabolic, anthropometric or biochemical parameters. The multilinear model included a variable combination of independent variables encompassing PWS status (Controls = 0; PWS = 1), age, sex, BMI SDS, US-derived NAFLD score, FLI, PMI, HOMA-IR, ALT, leptin and adiponectin levels. β coefficients and related significance values obtained from the models are reported. *P* < 0.05 was considered as statistically significant.

## Results

### Assessment of metabolic homeostasis

A summary of anthropometric and biochemical data is reported in Tables 1 and 2. BMI SDS values were comparable between the two groups and ranged, collectively, between 1.1 and 5.7. Among patients with PWS, 16 were obese including 4 with OSAS and, overall, 16 were prepubertal and 12 were postpubertal. Among controls, 20 were obese and, overall, 17 were prebubertal and 11 were postpubertal. None of the patients in the control group were diagnosed with OSAS. Abnormal glucose metabolism was detected in 2 children with PWS (7.1%) and 4 controls (14.3%): impaired glucose tolerance was found in 2 patients with PWS and 3 controls, impaired fasting glucose was found in 1 subject of the control group control and no patient with PWS. No cases of type 2 diabetes mellitus (T2DM) were diagnosed in either groups.

**Table 1:** Summary of anthropometric data obtained in patients with PWS and controls. Data are expressed as mean ± SD

Variables	PWS (*n* = 28)	Controls (*n* = 28)	*p*-value
Males/females	17/11	13/15	0.29
Prepubertal/pubertal	16/12	17/11	0.86
Age (years)	12.3 ± 3.1	13.4 ± 2.8	0.12
Weight (kg)	52.6 ± 15.1	77.0 ± 17.5	< 0.0001
Height (cm)	140.1 ± 12.5	158.0 ± 12.4	< 0.0001
Waist (cm)	81.0 ± 15.1	98.6 ± 11.1	< 0.0001
WHR	0.88 ± 0.10	0.94 ± 0.07	0.02
BMI SDS	2.5 ± 1.1	2.8 ± 0.5	0.26
FM (%)	45.0 ± 7.0	42.8 ± 6.1	0.31
FM (Kg)	26.3 ± 9.8	32.1 ± 10.4	0.08
Trunk fat (kg)	11.1 ± 4.8	14.2 ± 5.8	0.07
FFM (%)	52.7 ± 6.6	54.4 ± 5.8	0.40
FFM (Kg)	30.4 ± 10.1	41.1 ± 12.8	0.006
FFM/FM ratio	1.22 ± 0.33	1.33 ± 0.47	0.39
Trunk fat/FM ratio	0.91 ± 0.07	0.96 ± 0.07	0.04

*For abbreviation: WHR *waist-to-hip ratio,* BMI SDS *body mass index standard deviation score,* FM *fat mass,* FFM *fat-free mass

Comparison between populations was performed by ANOVA test. Significant differences are shown in bold characters

As shown in Table 1, anthropometric parameters differed between groups as subjects with PWS showed lower values of FFM (*p* < 0.01), trunk fat/FM ratio (*p* < 0.05), WC (*p* < 0.0001) and WHR (*p* < 0.05) with respect to controls.

**Fig. 1:**
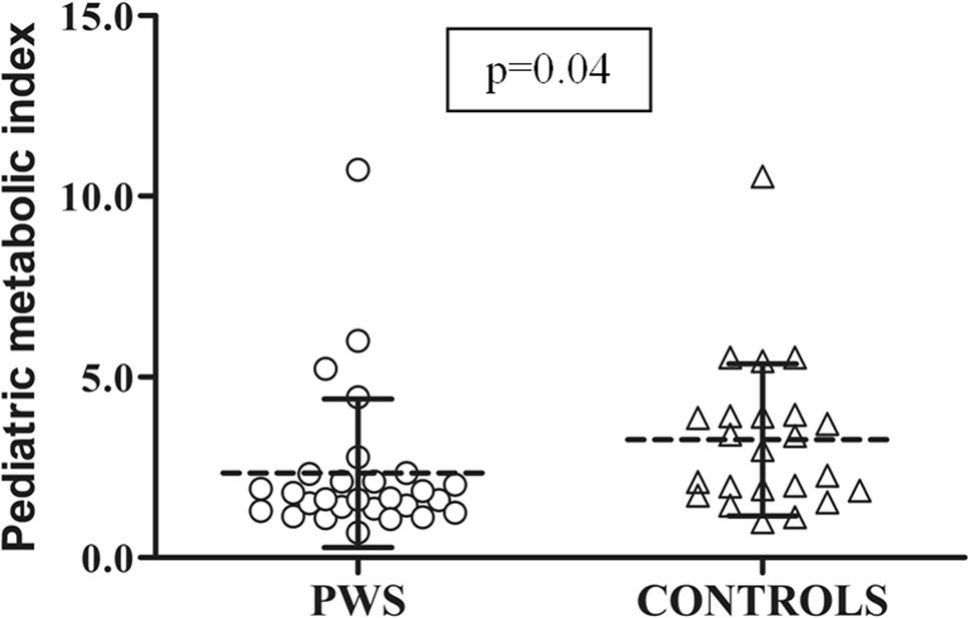
Individual PMI values in patients with PWS and controls. Dashed lines represent means and continuous lines represent standard deviation values in each population. ANOVA was used to evaluate the difference in PMI values between the 2 groups. PWS patients harbor significant lower PMI values compared to controls

Despite the less favourable body composition, children and adolescents with PWS harboured lower insulin and C-peptide levels, better insulin resistance, as well as higher adiponectin and HDL cholesterol levels, which were overall suggestive of a healthier metabolic profile compared to their controls (Table 2). This pattern was paralleled by sub-group profiles of PMI scores (Fig. 1).

**Table 2:** Summary of biochemical data obtained in patients with PWS and controls. Data are expressed as mean ± SD

Variables	PWS (*n* = 28)	Controls (*n* = 28)	*p*-value
Glucose OGTT_0_ (mg/dL)	83.1 ± 7.2	84.1 ± 8.3	0.63
Glucose OGTT_120_ (mg/dL)	102.6 ± 25.0	110.1 ± 27.1	0.30
Insulin OGTT_0_ (mIU/L)	10.1 ± 4.9	22.1 ± 14.1	< 0.0001
Insulin OGTT_120_ (mIU/L)	35.2 ± 17.2	134.7 ± 100.3	0.002
C-peptide (μg/L)	1.41 ± 0.55	2.33 ± 1.01	< 0.0001
HbA1c (%)	5.2 ± 0.3	5.0 ± 0.4	0.13
HOMA-IR	2.1 ± 1.1	4.7 ± 3.2	< 0.0001
CHO (mg/dL)	165.3 ± 29.7	161.8 ± 32.7	0.67
LDL CHO (mg/dL)	94.3 ± 18.3	94.9 ± 29.7	0.94
HDL CHO (mg/dL)	53.1 ± 10.9	45.9 ± 10.6	0.02
TG (mg/dL)	84.9 ± 53.4	100.9 ± 43.5	0.22
AST (U/L)	24.5 ± 7.5	25.0 ± 10.6	0.84
ALT (U/L)	22.7 ± 15.6	28.6 ± 22.7	0.27
GGT (U/L)	14.9 ± 5.9	16.3 ± 7.9	0.45
ANGPTL8 (ng/mL)	0.50 ± 0.29	0.48 ± 0.20	0.73
Leptin (ng/mL)	30.6 ± 18.5	31.4 ± 16.2	0.87
Adiponectin (µg/mL)	16.2 ± 7.8	9.2 ± 4.6	< 0.0001
PMI	2.3 ± 2.1	3.3 ± 2.1	0.04
FLI	22.2 ± 26.0	47.4 ± 23.9	0.001

For abbreviation: *OGTT* oral glucose tolerance test, *OGTT0 and OGTT120* OGTT at 0 and 120 min, *HbA1c* glycated haemoglobin, *HOMA-IR* homeostatic model of insulin resistance, *CHO* total cholesterol, *LDL CHO* low density lipoprotein cholesterol, *HDL CHO* high density lipoprotein cholesterol, TG triglycerides, *AST* aspartate aminotransferase, *ALT* alanine aminotransferase, *GGT* gamma glutamyl transpeptidase, *PMI* pediatric metabolic index, *FL*I fatty liver index

Comparison between populations was performed by ANOVA test. Significance is shown in bold characters

### Analysis of ANGPTL8 and indices of NAFLD

**Fig. 2:**
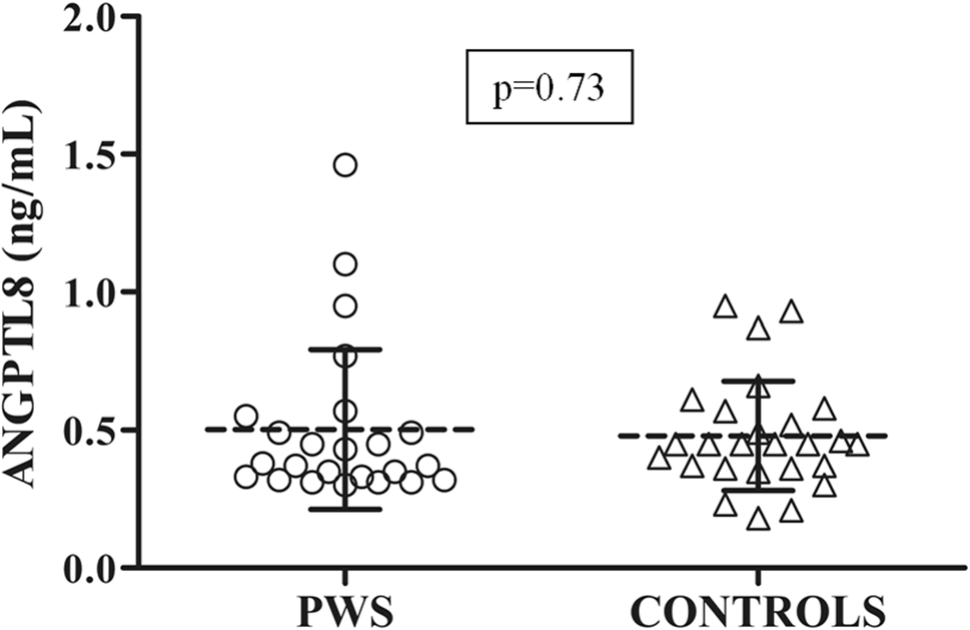
Individual values of circulating ANGPTL8 levels obtained in patients with PWS and controls. Dashed lines represent mean and continuous line represent standard deviation values in each population. ANOVA was used to evaluate the difference in ANGPTL8 values between the 2 groups. ANGPTL8 levels are similar between PWS and control group

Analysis of circulating ANGPTL8 showed measurable levels in all cases. As illustrated in Fig. 2, the distribution of ANGPTL8 levels was somewhat more dispersed around the mean in patients with PWS than controls owing to a limited number of outliers, which slightly increased the overall mean ANGPTL8 value. These four patients with PWS outliers were distinguished by higher severity of NAFLD (two had G2 and two had G3 degree of NAFLD score) and sex (3 males and 1 female). After removal of these outliers, however, ANGPTL8 levels remained similar between groups (*p* = 0.26). Among PWS patients with OSAS, only an outlier in ANGPTL8 levels was observed. Bearing in mind the limits due to the low sample size, no significant differences were found in ANGPTL8 levels and NAFLD grading between patients with and without OSAS (data not shown).

**Fig. 3:**
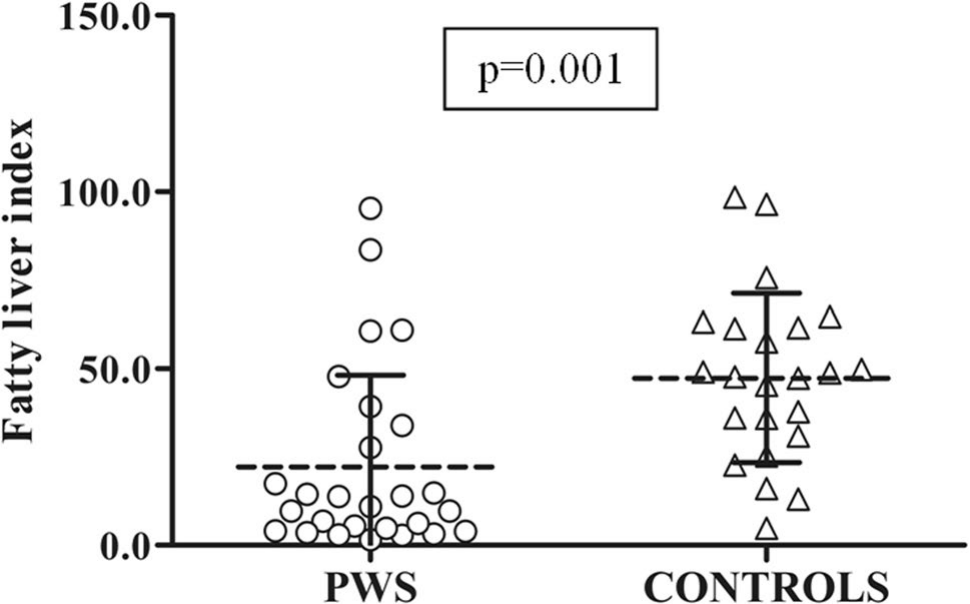
Individual values of FLI values obtained in patients with PWS patients and controls. Dashed lines represent means and continuous line represent standard deviation values in each population. ANOVA was used to evaluate the difference in FLI values between the 2 groups. PWS patients harbor significant lower FLI values compared to controls

As summarized in Table 2, liver function tests were similar between groups. However, FLI scores were significantly less severe in patients with PWS than controls (Fig. 3).

**Fig. 4:**
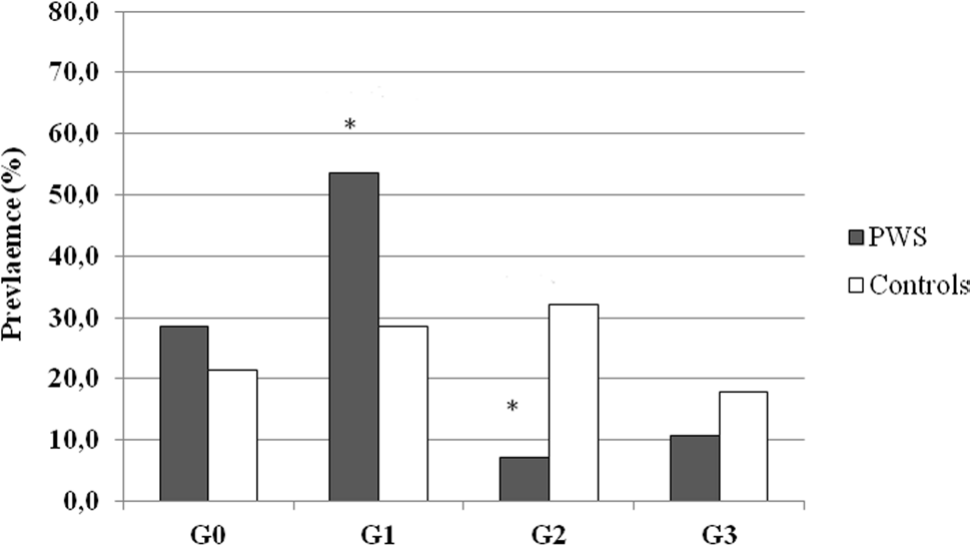
Prevalence (%) of NAFLD grading scores in patients with PWS and controls. The set level of significance is expressed as obtained by Chi square and summarized in the text. PWS patients show a higher prevalence of low NAFLD degrees, while exhibiting a lower prevalence of high NAFLD degrees when compared to controls

In terms of prevalence, the rate of US-derived NAFLD did not seem to differ between groups, since a US-derived pattern suggestive of NAFLD could be documented in 15 subjects with PWS and 19 controls (53.6% vs. 67.9%; χ^2^ = 1.20, *p* = 0.27). However, when NAFLD was stratified by grading, its severity appeared to be milder in patients with PWS than controls (Fig. 4). Specifically, patients with PWS showed a higher prevalence of low NAFLD degrees, i.e. G0 (28.6% vs. 21.4%) and of G1 (53.6% vs. 28.6%; χ^2^ = 3.72, *p* < 0.05), while exhibiting a lower prevalence of high NAFLD degrees, i.e. G2 (7.1% vs. 32.1%; χ^2^ = 5.54, *p* = 0.01) and G3 (10.7% vs. 17.9%) when compared to controls.

When study participants were grouped by the pubertal stage, no intra- and inter-group differences were found in ANGTPL8 levels and NAFLD prevalence. Likewise, there were no differences in ANGPTL8 and NAFLD when patients with PWS were analysed according to genotype and/or GH treatment (data not shown).

### Correlation analyses

Correlation analysis were performed in the two groups as a whole and then in separate groups. Analysis on merged data (Table 3) only showed positive associations between ANGPTL8 and BMI SDS (*r* = 0.44, *p* < 0.01), leptin levels (*r* = 0.35, *p* < 0.05) and HbA1c (*r* = 0.35, *p* < 0.05).

**Table 3:** Pearson’s correlation analysis between ANGPTL8 levels and anthropometric and biochemical parameters in the study populations as a whole

Parameters	ANGPTL8 levels	
	*r*	*p*-value
Age (years)	– 0.11	0.46
Gender	– 0.02	0.91
Pubertal stage	– 0.18	0.22
Status (PWS or controls)	0.05	0.73
BMI SDS	0.44	0.002
FM (kg)	0.08	0.67
FFM (kg)	– 0.01	0.94
Glucose OGTT_0_ (mg/dL)	0.02	0.90
Glucose OGTT_120_ (mg/dL)	0.12	0.41
Insulin OGTT_0_ (mIU/L)	0.08	0.59
Insulin OGTT_120_ (mIU/L)	0.26	0.23
HbA1c (%)	0.35	0.013
HOMA-IR	0.06	0.70
PMI index	0.26	0.23
ALT (U/L)	0.23	0.10
NAFLD (US)	0.20	0.17
FLI index	0.25	0.10
IGF-1 (ng/mL)	0.23	0.20
Leptin (ng/mL)	0.35	0.013
Adiponectin (µg/mL)	– 0.20	0.89

For status: PWS = 1, controls = 0; for gender: male = 1, female = 2. Significance is shown in bold characters. For abbreviation: *BMI SDS*, body mass index standard deviation score; *FM*, fat mass; *FFM*, fat free mass; *PMI*, paediatric metabolic index, OGTT0 and OGTT120, OGTT at time 0 min and 120 min, HbA1c glycated haemoglobin, HOMA-IR homeostatic model of insulin resistance, *ALT* alanine aminotransferase, *NAFLD* non-alcoholic fatty liver disease, *FLI* fatty liver index, *IGF-1* insulin-like growth factor-1

**Fig. 5:**
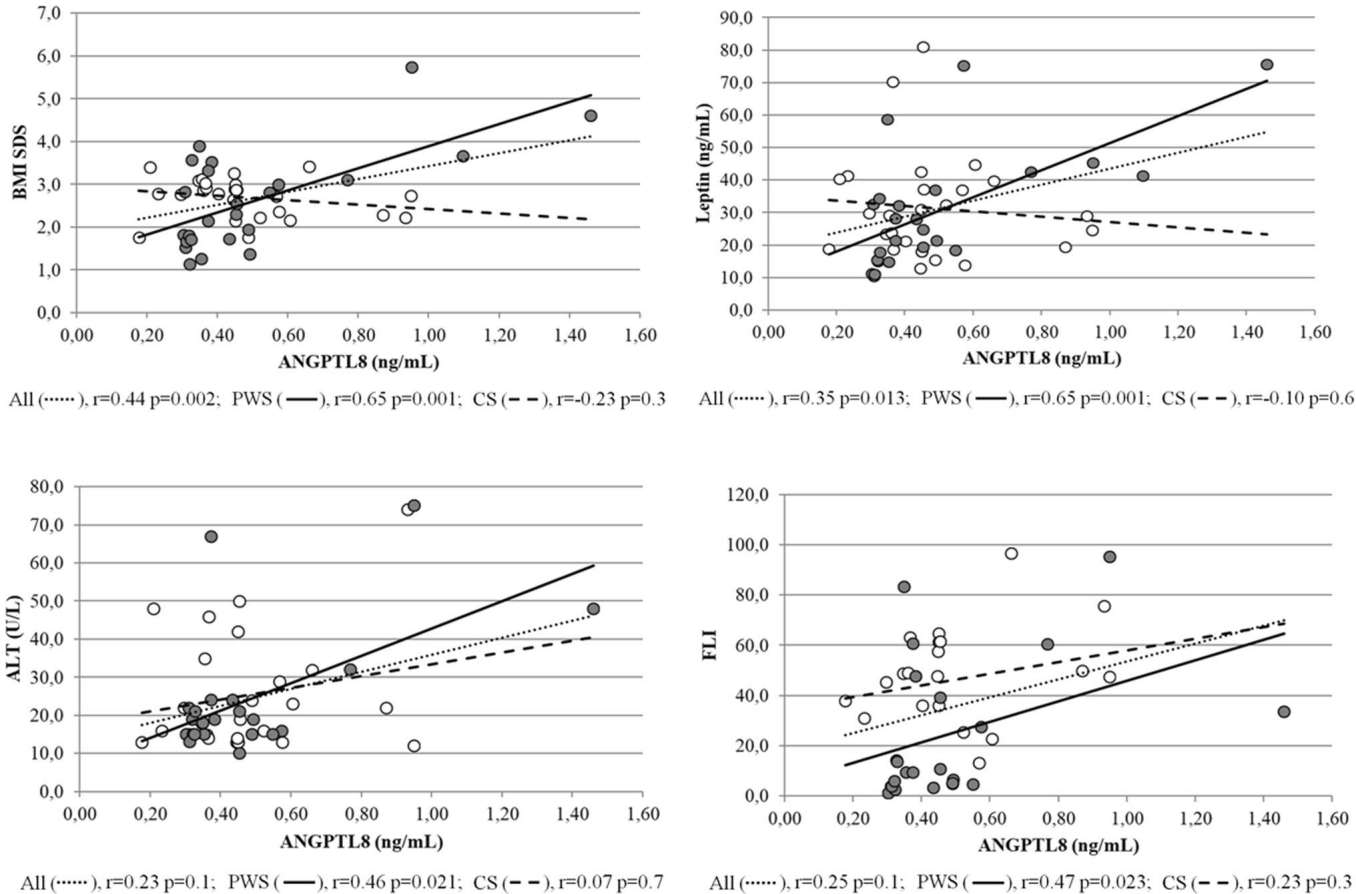
Bivariate correlation analysis between ANGPTL8 levels and BMI SDS, leptin, ALT and FLI. Closed circles: PWS; open circles: controls. Correlation coefficients and significance are displayed at the bottom of each plot. Associations revealed statistical significance only in children and adolescents with PWS

**Fig. 6:**
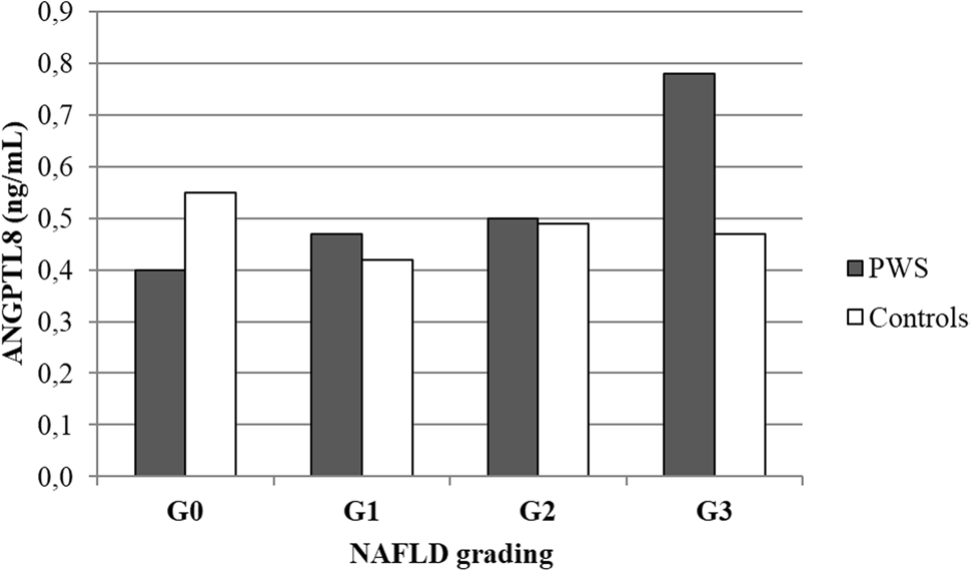
Histogram illustrating ANGPTL8 levels grouped by NAFLD grading in the 2 populations. PWS patients show increasing levels of ANGPTL8 across the different degrees of NAFLD

Associations were subsequently explored in individual groups and revealed statistical significance only in children and adolescents with PWS between ANGPTL8 levels and BMI SDS (*r* = 0.65, *p* = 0.001), leptin (*r* = 0.65, *p* = 0.001) ALT (*r* = 0.46, *p* < 0.05), FLI (*r* = 0.47, *p* < 0.05) (Fig. 5), as well as prevalence (*r* = 0.58, *p* < 0.01) and grading of NAFLD (*r* = 0.42, *p* < 0.05). Figure 6 showed ANGPTL8 levels grouped by NAFLD grading in the 2 populations. These associations remained significant after controlling for age and sex. Oppositely, ANGPTL8 levels were unrelated to any metabolic and liver-associated variables in the control group.

Moreover, an association was found between FLI scores and NAFLD grading (All: *r* = 0.52, *p* < 0.0001; PWS: *r* = 0.53, *p* < 0.01; controls: *r* = 0.43, p < 0.05), as well as with trunk fat mass in the population as a whole and in PWS group (All: *r* = 0.60,* p* < 0.0001; PWS: *r* = 0.70, *p* = 0.001, Controls: *r* = 0.43, *p* = 0.07). With regards to other metabolic measures, an association related FLI scores to PMI values (All: r = 0.57, p < 0.0001; PWS: *r* = 0.49, *p* = 0.01; Controls: *r* = 0.62, *p* < 0.01). Only in patients with PWS an association occurred between PMI and ALT (*r* = 0.48, *p* < 0.01) and the presence of NAFLD (*r* = 0.45, *p* < 0.01).

Stepwise multivariable regression analysis was performed in the two groups as a whole, and a number of models were explored to analyse the predictors of ANGPTL8 levels in the study populations. Analysis documented that ANGPTL8 levels were only predicted by BMI SDS (standardized β = 0.49, *p* = 0.001 for both). After removal of BMI SDS from the regression equation, leptin levels (standardized β = 0.66, *p* = 0.001) and the presence of NAFLD (standardized β = 0.57, *p* = 0.006) acted as independent predictors of ANGPTL8 levels. Results were similar when this regression model was repeated in the group of patients with PWS. No predictor entered the regression equation in controls.

## Discussion

The present study analysed the association between ANGPTL8 levels and adiposity, metabolic profile and NAFLD in relation to PWS status in children and adolescents. Our results show that ANGPTL8 levels are comparable between patients with PWS and controls and that, in PWS, this liver-derived protein is closely associated with the presence and severity of NAFLD.

It has long been shown that PWS predisposes to a peculiar somatic and metabolic phenotype that is featured by short stature, peripheral adiposity, sarcopenia and abnormalities in pituitary hormones [33, 34]. This unfavourable setting predisposes patients with PWS to develop typical obesity-related traits, yet their metabolic impairment results milder when compared to controls matched for BMI [13, 33–36]. In fact, children and adolescents with PWS rarely develop dyslipidaemia [17] and harbour a milder degree of hyperinsulinaemia and insulin resistance than controls matched for BMI [36–39]. Although the precise mechanisms underlying this metabolic paradox remain unclear, some authors suggested that orexigenic hormones like ghrelin [40] or insulin-sensitive adipokines like adiponectin could intervene to diminish the consequences of the abnormal body composition of subjects with PWS [34, 39], while others hypothesized a decrease in vagal parasympathetic tone to the pancreas as a potential cause of reduced insulin secretion [8]. Further, specific patterns in pro-inflammatory molecules [41] and microbial taxa exist in PWS [42]. This metabolic peculiarity is here confirmed, as we found healthier values of insulin and C-peptide, insulin resistance, HDL cholesterol and adiponectin in patients with PWS despite lower values of FFM, when compared to controls matched for BMI SDS. Likewise, analysis of PMI, a predictor of metabolic diseases in paediatric populations [31], showed lower values in patients with PWS than controls, along with its correlation with ALT levels and NAFLD in PWS. As such, this result suggests a potential role for PMI as a surrogate marker of liver steatosis.

Liver is a target organ of the metabolic syndrome associated with obesity. Attention has recently focused on liver- and adipose tissue-produced adipokine ANGPTL8, which is involved in different metabolic pathways relating to glucose and lipid metabolism as well as liver function [20, 21]. Its activity involves the ability of reducing serum triglyceride clearance and improving insulin resistance through the regulation of hepatic and peripheral insulin sensitivity by yet unclear mechanisms [22].

Based on these evidences, the current study was undertaken to investigate circulating ANGPTL8 levels for its ability to predict NAFLD in young patients with PWS and their controls. ANGPTL8 was measurable in all sera and its levels appeared to parallel those previously described in a large paediatric group [43]. Despite a wider interindividual variability of ANGPTL8 levels in patients with PWS than controls, its levels did not differ between populations and were significantly correlated with BMI SDS. This result agrees with previous findings showing that ANGPTL8 levels increase with obesity in paediatric ages [44]. On the other hand, ANGPTL8 was unrelated to lipids, glucose homeostasis, insulin and insulin resistance. This dissociation corroborates previous results obtained in adults affected by obesity with and without PWS [18], and agrees with the inference proposed by Cox et al. who suggested that ANGPTL8 is not as robustly involved in β-cell proliferation as originally proposed [45]. Hence, although experimental evidence suggests that ANGPTL8 is involved in circadian liver response to food intake, glucose tolerance, insulin resistance and ectopic lipid accumulation [46–48], our results seem to imply that ANGPTL8 is not responsible for the lower levels of insulin seen in patients with PWS in comparison to controls matched for BMI SDS.

Noteworthy, previous studies described higher ANGPTL8 mRNA expression in omental fat from individuals affected by obesity with NAFLD and insulin resistance as compared with controls matched for BMI with a normal insulin sensitivity [48]. In our study, liver function tests and prevalence of NAFLD were similar between patients with and without PWS, yet children and adolescents with PWS exhibited significantly lower values of FLI and less severe NAFLD scores than controls. Importantly, ANGPTL8 levels correlated with markers of NAFLD and, in single-group analyses, a reasonable correlation between NAFLD and circulating ANGPTL8 emerged only in subjects with PWS. Whether this divergent behaviour reflects a different metabolic milieu existing in the two study populations remains unclear. Nevertheless, it is interesting to note that a previous study found strong positive associations between ANGPTL8 and metabolic markers in individuals without insulin resistance but not in patients with T2DM [49]. In this context, it is necessary to point out that limitations exist in correctly assessing NAFLD in children and adolescents by means of non-invasive methodologies [50], and the indirect approach used herein harbours caveats that may have hampered the significance of our observations [51]. However, while liver biopsy is the gold-standard method for the accurate staging of NAFLD, several previous studies demonstrated a strong correlation between US findings and the presence as well as the degree of NAFLD documented by biopsy [28, 52, 53].

Based on these few evidences, we speculate that ANGPTL8 could act as an (healthy) hepatokine or (unhealthy) adipokine depending on the metabolic milieu, such that the correlation between ANGPTL8 and NAFLD seen in patients with PWS reflects a healthier metabolic status compared to controls matched for BMI SDS. If this holds true, a dysmetabolic progression occurring during adulthood could disentangle ANGPTL8 function as an adipokine, which could then act to signal comorbidities relating to insulin resistance and NAFLD [18]. This hypothesis awaits verification.

In conclusion, ANGPTL8 levels are similar in PWS and controls and, overall, they are directly associated with the presence and severity of NAFLD in patients with PWS. Further studies should investigate the potential genetic basis of these findings.

## Data Availability

The datasets generated during and/or analysed during the current study are available from the corresponding author on reasonable request.
